# Effect of Line-Magnet Stimulation on HRV: A Double-Blind, Randomized, Crossover Trial

**DOI:** 10.3390/healthcare9040421

**Published:** 2021-04-05

**Authors:** Han-Gue Jo

**Affiliations:** School of Computer Information and Communication Engineering, Kunsan National University, Gunsan 54150, Korea; hgjo@kunsan.ac.kr; Tel.: +82-63-469-4694

**Keywords:** heart rate variability, meridian, line-magnet

## Abstract

The acupuncture point mapped into the body meridian line has been the subject of extensive research; however, the scientific basis of the body meridian line remains unknown. In the present study, a pair of line-magnets was attached along the heart meridian line between HT4 and HT6 (intervention), and heart rate variability (HRV) was examined in a double-blind, randomized, crossover trial. Forty-five healthy young adults were randomly assigned into two groups and received two interventions in a different order. The only difference between the interventions was that the magnet pole was swapped. The results showed that the frequency-domain (LF, LF/HF) properties of HRV, which reflects underlying autonomic nervous system activity, changed when the magnetic pole’s direction along the heart meridian line was changed. This finding suggests that a line-magnet attached along to the heart meridian line may affect the cardiovascular system.

## 1. Introduction

According to traditional oriental medicine, body-meridian lines are pathways, conduits, or channels that run through the human body and life energy; *qi*, flows along these meridians [1,2]. The existence of the body-meridian line in humans remains a mystery; however, specific acupuncture points along the body-meridian line are an accepted form of traditional, complementary and alternative medicine (TCAM) for various conditions. For example, needling or pressing the skin in different directions can cause different effects: directing the stimulus with or against the meridian line is performed to achieve reinforcement or reduction, respectively [3]. Accordingly, several studies have reported the body meridian’s electrical nature, suggesting a preferential orientation of electric energy flow along the body-meridian line [4,5,6,7].

Few studies have assessed the effect of line-magnets (see the Section 2) to trigger the body-meridian line on circadian rhythms and brain activity [8,9]. A pair of line-magnets attached along the heart meridian (HT) line may change heart rate variability (HRV) [8] and electroencephalogram patterns [9] when the direction of the magnetic pole is swapped. However, the study design limitations of previous studies, including the “order” effect (wherein the order in which more than one task condition are administered may affect the results), have prevented a definitive conclusion regarding the scientific basis of the body-meridian line. It should be noted that although HT is thought to originate in the heart organ [3], the meridian names do not necessarily represent the same organ names as in Western science and medicine [2].

The present study aimed to examine the effect of line-magnets on HRV in a double-blind, randomized, crossover trial. It was hypothesized that stimulating the HT using a line-magnet may change the HRV depending on the magnetic pole in which they align [8]. In particular, the ratio between the low and high frequency components of HRV, which represent the balance between the sympathetic and parasympathetic nervous system [10] (but see also a recent review [11]), may be changed depending on the direction of the line-magnet pole [8].

## 2. Materials and Methods

**Figure 1:**
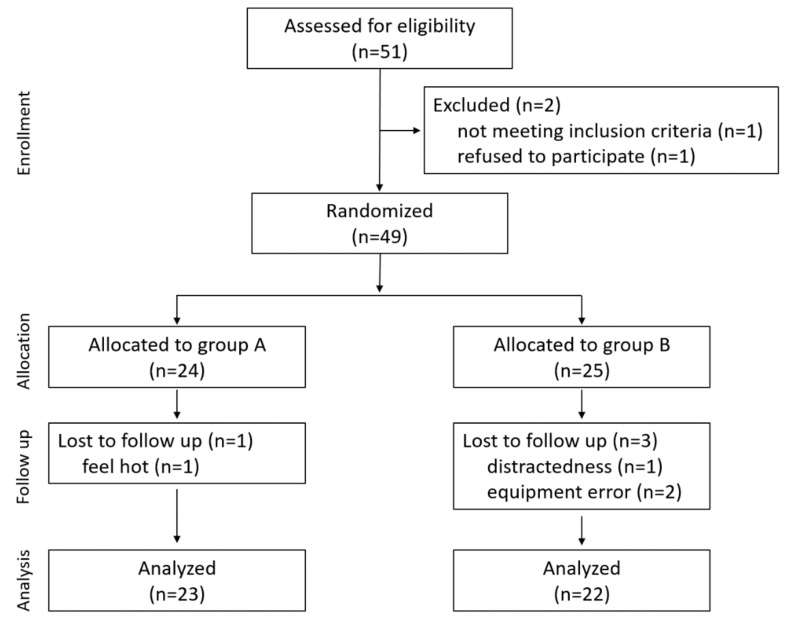
Participant flow diagram.

Fifty-one healthy young adults between 19 and 26 years of age (mean ± SD age: 20.18 ± 0.95; 37 females) volunteered for the present study. Participants were requested to abstain from smoking, alcohol, and caffeinated drinks for at least twelve hours before the experiment. No medical history related to cardiovascular disease was reported. Written informed consent was collected from all participants after the procedure was fully explained. The institutional review board of Kongju National University approved this study. Participants were randomly allocated to one of two groups using a computer-generated randomized schedule. One participant was excluded based on eligibility criteria, and one refused to participate (Figure 1). Four volunteers were excluded for the following reasons: two for equipment error, one for distractedness, and one for feeling hot. Of the 45 participating volunteers, 23 were randomly assigned to group A (18 females) and 22 to group B (14 females). The required sample size was forty-one for this matched pairs design when the effect size (d), α level, and power (1 − β) are 0.45, 0.05, and 0.8, respectively.

**Figure 2:**
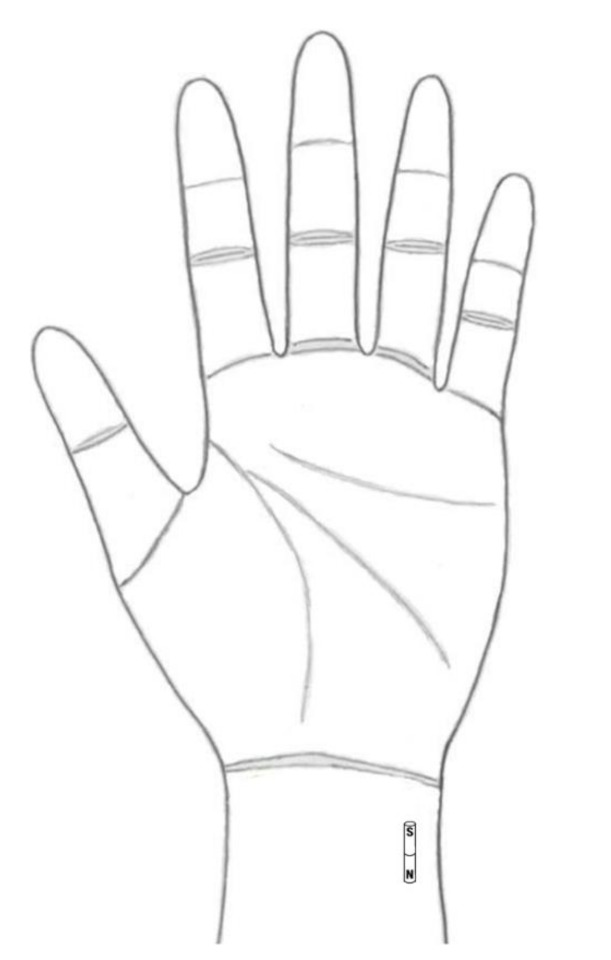
A pair of line-magnets is placed in line along the heart meridian. The line-magnets are positioned with N poles proximal and S poles distal (modified from [8]).

Two nickel-plated line-magnets were used to stimulate the HT. These were cylinders (1.5 × 5 mm NEO-MAG (Nd-Fe-B)) with a manufacturer rating of 2600 Gauss (mean surface field strength) (Figure 2). Using adhesive tape, the two line-magnets were attached in line along the HT between HT4 and HT6 on the wrists of both arms [12]. The same pole of all line-magnets was pointed by a black color on one side of the tip of the line-magnets (they repel each other), but the magnetic pole was double-blind throughout the experiments. The participants and experimenters were unaware of who received which intervention. The magnetic pole was confirmed using a compass needle after data collection and statistical analysis had been completed. The black painted pole of the line-magnets was confirmed to attract the N pole of the compass needle.

**Figure 3:**
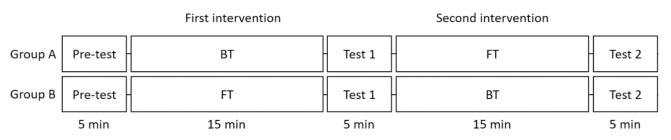
Data collection process. Groups A and B underwent two different interventions in a different order. FT intervention: the line-magnets were positioned with N poles proximal and S poles distal, in which the N pole of the line-magnet was attached to the direction of the starting point of the heart meridian [8]. BT intervention: the N pole of line-magnet was attached to the direction of the end point of the heart meridian.

All participants received the same interventions but in a different order. A pair of line-magnets were attached to the HT for fifteen minutes in different directions (first and second interventions; Figure 3). Each intervention was followed by a five minute test period without the line-magnets (Tests 1 and 2). The first intervention for participants in group A was attaching the black point of the magnet pair in the direction of the starting point of the HT (BT, backward treatment as named in the previous study [8]) and vice versa for the second intervention (FT, forward treatment). The first intervention for participants in the group B was attaching as the black point of the magnet pair in the direction of the end point of the HT (FT) and vice versa for the second intervention (BT). In brief, group A received BT followed by FT, while group B received FT followed by BT for the first and second interventions, respectively.

Electrocardiogram (ECG) was measured with a sampling rate of 512 Hz during Tests 1 and 2 without line-magnets. To ensure baseline equality and the absence of statistically significant differences between the study conditions, baseline activity before the first intervention was recorded for five minutes (Pre-test). The ECG was recorded in a sitting position with relaxed arms. Before measurement began, participants had over twenty minutes of rest. In total, the experiment took approximately 65 min. During the recordings, participants were asked to maintain their normal breathing in a comfortable sitting position. The experiment was performed in a quiet laboratory room at the Kongju National University. The room temperature was 23.14 ± 0.73 °C (mean ± SD).

MatLab (MathWorks, Inc., Natick, MA, USA) and Kubios HRV [13] were used to identify the R-peaks on ECG after preprocessing, which included band-pass filtering (to reduce frequency components of no interest), squaring of the data samples (to highlight R-peaks), and moving average filtering (to smooth close-by peaks) [14]. Thereafter, the RR intervals were subjected to time-domain, frequency-domain, and non-linear assessments. For the time-domain analysis, the mean heart rate (mHR), standard deviation of RR intervals (SDNN), root mean square difference of successive RR intervals (RMSSD), and portion of adjacent variation of >50 ms (pNN50) were calculated. The SDDN index quantifies the total amount of variability that is influenced by changes in the sympathetic and parasympathetic activity, and the RMSSD and pNN50 indices quantify successive beat to beat RR interval differences and represent parasympathetic activity [10,15,16]. The frequency-domain components were estimated using a Fast Fourier transformation (FFT) and the power were determined for very low (VLF, 0–0.04 Hz), low (LF, 0.04–0.15 Hz), and high (HF, 0.15–4 Hz) frequencies. The LF/HF ratio was also calculated. The HF corresponds to the performance of the vagus nerve on the heart and the LF represents the joint action of the vagal and sympathetic components with a predominance of the sympathetic ones [10,15,16]. Therefore, the LF/HF ratio reflects the balance between the sympathetic and parasympathetic components of the autonomic nerve system (but also see a recent review [11]). The VLF has not been well defined; however, its modulation is attributed to sympathetic activity [10]. The non-linear properties of RR intervals were evaluated using the Poincare plot method, which includes the standard deviation of data from the long axis of the Poincare plot (SD1), the standard deviation of the data from the axis perpendicular to the long axis of the Poincare plot (SD2), and SD1/SD2 ratio. SD1 is an index of instantaneous beat-to-beat variability and SD2 represents long-term HRV. Therefore, SD1/SD2 is the ratio between the short- and long-term variations of the RR intervals.

Statistical analysis was conducted in two stages. First, a two-tailed paired t-test was performed for within-individual comparison. Individual data measured after the BT (Post-BT) was compared with the data measured after the FT (Post-FT). Second, to supplement the within-individual comparison, a repeated-measures ANOVA was performed with group (A and B) as the between-subject factor and time (Pre-test, Test 1, and Test 2) as the within-subject factor. If the data violated the assumption of sphericity, a Greenhouse–Geisser p-value was presented. A post-hoc two-tailed t-test was applied to address differences in one measure point between groups. All values are reported as mean ± standard error of the mean unless mentioned otherwise.

## 3. Results

The baseline characteristics of the two groups did not differ in age (A, 20.30 ± 0.05; B, 20.09 ± 0.03; *p* = 0.482) and time when the experiment was conducted (hour; A, 14.72 ± 0.16; B, 15.00 ± 0.14; *p* = 0.782). Baseline HRV was not significantly different between groups (*p* > 0.102 for all).

The two-tailed paired *t*-test revealed a significant effect of intervention on the SDNN in the time-domain analysis, LF and LF-HF ratio in the frequency-domain analysis, and the SD2 and SD1/SD2 ratio in the non-linear analysis (Table 1). All parameters, except SD1/SD2, were increased at Post-BT when compared with Post-FT. However, only the LF and LF/HF ratio survived after Bonferroni correction for multiple comparisons.

A repeated-measures ANOVA revealed a significant main effect of time on mHR, SDNN, VLF, LF, SD2, and SD1/SD2 (Table 2). The mHR has significantly decreased from the Pre-test phase (78.76 bpm ± 1.35) to Test 1 (76.89 bpm ± 1.31, corrected *p* = 0.003). Test 2 (77.31 bpm ± 1.31) was not significantly different from the Test 1 (corrected *p* ≈ 1.000); however, a significant trend from Pre-test was found (corrected *p* = 0.056). The same pattern of results was observed in SDNN (Pre-test = 48.64 ms ± 2.55; Test 1 = 57.02 ms ± 2.87; Test 2 = 58.40 ms ± 2.53), VLF (Pre-test = 949.98 ms^2^ ± 91.46; Test 1 = 1469.10 ms^2^ ± 195.72; Test 2 = 1379.33 ms^2^ ± 143.31), LF (Pre-test = 720.43 ms^2^ ± 93.34; Test 1 = 966.07 ms^2^ ± 107.18; Test 2 = 870.57 ms^2^ ± 77.46), SD2 (Pre-test = 63.57 ms ± 3.18; Test 1 = 75.56 ms ± 3.82; Test 2 = 77.72 ms ± 3.34), SD1/SD2 (Pre-test = 0.39 ± 0.02; Test 1 = 0.36 ± 0.02; Test 2 = 0.35 ± 0.02). These results indicated that the significant main effect of time is mainly due to a difference between the baseline activity (Pre-test) and study conditions (Test 1 and Test 2).

Notably, the repeated measures ANOVA revealed a significant time × group interaction effect on SDNN, LF, LF/HF, SD2, and SD1/SD2. The greater statistical significance of the time × group interaction effect relative to the time effect was observed in the LF and LF/HF ratio. Since the group difference of the study design was the order of the task conditions, BT and FT (Figure 2), the change from the Test 1 to Test 2 was calculated and compared between groups (FT–BT for group A and BT–FT for group B). This revealed a significant group difference in SDNN (A: −3.17 ms ± 0.67, B: 5.94 ms ± 0.50, *p* = 0.028), LF (A: −380.72 ms^2^ ± 27.11, B: 189.72 ms^2^ ± 20.98, *p* = 0.001), LF/HF (A: −0.36 ± 0.38, B: 0.94 ± 0.07, *p* = 0.002), SD2 (A: −4.46ms ± 0.92, B: 8.78ms ± 0.71, *p* = 0.021), and SD1/SD2 (A: 0.012 ± 0.03, B: −0.042 ± 0.003, *p* = 0.011), which are in accordance with the results of the within-individual comparison.

## 4. Discussion

This double-blind, randomized, crossover trial examined HRV while participants had a pair of line-magnets attached to sections of the HT between HT4 and HT6. The frequency-domain properties of the HRV were significantly changed depending on the direction of the magnet pole.

Previous studies have reported that line-magnet stimulation on the HT alters brain activity and HRV components [8,9]. The mRH is reduced and the LF and LF/HF ratio are increased during BT when compared with FT [8]. In line with this, the within-individual comparison showed a significant increase in LF and LF/HF ratio after BT when compared with FT. However, it did not show a significant change in mHR. On the other hand, there was a significant increase in SDNN after BT when compared with FT. SDNN is associated with both the parasympathetic and sympathetic nervous system and it is highly correlated with VLF and LF rhythms (Pre-test: r = 0.764, 0.840, respectively; Test 1: r = 0.688, 0.852, respectively; Test 2: r = 0.629, 0.664, respectively) [10,17].

Frequency-domain analysis provides an index of autonomic function [17,18,19]. Low-frequency (LF) oscillations refer to both parasympathetic and sympathetic activities, while high-frequency (HF) oscillations refer to parasympathetic activity. The ratio of LF to HF may estimate the balance between sympathetic and parasympathetic nervous system activity. In this model, a high LF/HF ratio reflects sympathetic dominance when the body engages in fight-or-flight behaviors. In contrast, a low LF/HR ratio indicates parasympathetic dominance when the body is relaxed and resting.

Differences in the LF and LF/HF ratio, but not the HF, survived the Bonferroni multiple testing correction and have been associated with the line-magnet stimulation in a previous study [8]. A high LF and LF/HF ratio was observed after BT, which was when the N pole of the line-magnet (attracting the S pole of the compass needle) was positioned in the direction of the end point of the HT. This suggests that BT may induce a higher sympathetic activity relative to the parasympathetic activity. However, this should be interpreted with caution because the LF/HF ratio is often affected by changes in the LF that can be easily confounded by respiration mechanisms [17,20,21]. Additionally, the LF/HF ratio remains controversial because interactions between the parasympathetic and sympathetic nervous system are complex, non-linear, and frequently non-reciprocal [22].

Electrical properties have been reported at acupuncture points over the past fifty years, and the meridian lines have been characterized by lower electrical impedance [4,5,6,7,23,24,25,26]. Nevertheless, the physiological characteristics of the body-meridian remain unclear. The present study further suggests that there are distinguishing magnetic properties of the body-meridian line. However, it is possible that the use of line-magnets may have partially affected the cardiovascular system directly and HRV changes might not be due to the stimulation of the HT. It should be noted that a previous study applying the same line-magnet stimulation on a control position close to the HT reported no significant change in HRV [8]. Further studies with a careful modification of the study paradigm, such as including a sham intervention using demagnetized metal, would further elucidate the magnetic properties of the meridian.

Another limitation of this study is that the 5-min washout period (Test 1 and Test 2) between interventions might be not the optimal duration required for subjects to return to their baseline HRV. The washout period for the line-magnet effect is unknown; therefore, it cannot rule out that the changes in HRV recorded during Test 2 might be affected by the first intervention. Nonetheless, it should be noted that the LF and LF/HR ratio demonstrated similar patterns when comparing groups recorded during Test 2 after the second intervention (i.e., the LF and LF/HR ratio decreased and increased at Test 2 in groups A and B, respectively). Further studies should introduce a variable washout period to determine the optimal time between interventions. In addition, respiration was not controlled in the present study. Previous studies have shown that HRV indices, in particular LF, are correlated with respiration rate [20,21]. In this study, it was unable to ascertain the influence of the respiration rate on HRV. Use with other physiological measures such as respiration, skin conductance, and blood pressure variability, may prove useful in extending the findings of the present study and further elucidate how sympathetic and parasympathetic activation are reflected in the LF and LF/HF ratio under line-magnet intervention. Lastly, in hindsight, both groups A and B showed a significant time effect, indicating a significant drop of mHR that was not expected, since all participants took more than 20 min of rest in a sitting posture before recordings. It should be noted that the time effect was mainly due to the difference between the pre-test and interventions. Further study is required to evaluate the importance of an adequate rest period for the effect of line-magnets on the cardiovascular system.

## 5. Conclusions

This double-blind, randomized, crossover trial suggested that a pair of line-magnets attached in line along to the heart meridian may affect HRV. Changes in HRV were associated with the direction of the magnetic pole. Stimulation on other sections of the heart meridian with a careful task design should be further investigated and will help to verify the current findings.

## Figures and Tables

**Table 1:** Within-individual comparison of the heart rate variability.

HRV	Post-FT	Post-BT	*p* Value	95% Confidence Interval of the Difference		Cohen’s d
	Mean ± SD	Mean ± SD		Lower	Upper	
mHR (bpm)	77.027 ± 0.193	77.185 ± 0.193	0.762	−1.21	0.89	0.045
SDNN (ms) *	55.374 ± 0.357	59.892 ± 0.449	0.029	−8.55	−0.50	0.338
RMSSD (ms)	37.731 ± 0.327	38.448 ± 0.361	0.536	−3.03	1.60	0.092
pNN50 (%)	18.345 ± 0.334	18.904 ± 0.342	0.630	−2.88	1.76	0.072
VLF (ms^2^)	1295.012 ± 22.724	1552.13 ± 27.637	0.193	−648.68	134.45	0.197
LF (ms^2^) **	774.7 ± 10.764	1062.04 ± 16.310	0.001	−453.37	−121.31	0.520
HF (ms^2^)	557.341 ± 8.646	608.045 ± 9.626	0.179	−125.45	24.04	0.204
LF/HF **	1.783 ± 0.024	2.426 ± 0.036	0.002	−1.03	−0.25	0.496
SD1 (ms)	26.717 ± 0.231	27.225 ± 0.255	0.536	−2.15	1.13	0.093
SD2 (ms) *	73.262 ± 0.474	79.485 ± 0.684	0.022	−12.14	−1.00	0.354
SD1/SD2 *	0.366 ± 0.002	0.339 ± 0.002	0.013	0.01	0.05	0.384

HRV, heart rate variability; mHR, mean heart rate; SDNN, standard deviation of RR intervals; RMSSD, root mean square difference of successive RR intervals; pNN50, portion of adjacent varying by >50 ms; VLF, very low frequency spectral analysis of RR intervals; LF, low frequency spectral analysis of RR intervals; HF, high frequency spectral analysis of RR intervals; SD1, standard deviation of data from the long axis of the Poincare plot; SD2, standard deviation of the data from the axis perpendicular to the long axis of the Poincare plot. *, *p* < 0.05; **, Bonferroni corrected *p* < 0.05.

**Table 2:** A repeated-measures ANOVA results on the heart rate variability.

HRV	F, *p*		Group A			Group B		
	Time	Time × Group	Pre-Test	Test 1	Test 2	Pre-Test	Test 1	Test 2
mHR (bpm) ^†^	6.392, 0.003	0.503, 0.928	79.30 ± 1.88	77.40 ± 1.83	77.65 ± 1.83	78.22 ± 1.92	76.38 ± 1.87	76.96 ± 1.88
SDNN (ms) ^†,‡^	16.640, <0.001	6.597, 0.002	49.46 ± 3.57	55.91 ± 4.01	52.74 ± 3.54	47.82 ± 3.65	58.13 ± 4.10	64.06 ± 3.62
RMSSD (ms)	1.517, 0.228	0.877, 0.394	34.91 ± 3.87	36.17 ± 3.29	34.83 ± 3.14	36.97 ± 3.69	40.77 ± 3.36	40.83 ± 3.21
pNN50 (%)	1.376, 0.257	0.721, 0.462	15.24 ± 3.56	16.43 ± 3.16	14.44 ± 3.11	18.95 ± 3.63	22.43 ± 3.23	21.49 ± 3.18
VLF (ms^2^) ^†^	4.869, 0.014	1.262, 0.288	1025.88 ± 127.90	1567.53 ± 273.69	1222.65 ± 200.41	874.07 ± 130.78	1370.66 ± 279.84	1536.02 ± 204.91
LF (ms^2^) ^†,‡^	3.640, 0.030	4.880, 0.010	696.87 ± 130.53	1111.03 ± 149.88	730.31 ± 108.32	743.99 ± 133.46	821.11 ± 153.24	1010.83 ± 110.76
HF (ms^2^)	0.396, 0.572	0.977, 0.344	635.14 ± 161.89	542.80 ± 85.31	449.76 ± 83.98	643.65 ± 165.52	669.81 ± 87.22	676.25 ± 85.87
LF/HF ^‡^	2.016, 0.139	4.572, 0.013	1.82 ± 0.32	2.31 ± 0.23	1.95 ± 0.34	1.83 ± 0.33	1.61 ± 0.23	2.55 ± 0.34
SD1 (ms)	1.519, 0.228	0.877, 0.394	24.72 ± 2.74	25.61 ± 2.33	24.66 ± 2.22	26.18 ± 2.80	28.87 ± 2.38	28.91 ± 2.27
SD2 (ms) ^†,‡^	18.294, <0.001	7.091, 0.002	65.15 ± 4.45	74.56 ± 5.35	70.10 ± 4.67	62.00 ± 4.55	76.57 ± 5.47	85.35 ± 4.78
SD1/SD2 ^†,‡^	6.514, 0.004	6.205, 0.005	0.36 ± 0.02	0.35 ± 0.02	0.36 ± 0.02	0.42 ± 0.02	0.37 ± 0.02	0.33 ± 0.02

HRV, heart rate variability; mHR, mean heart rate; SDNN, standard deviation of RR intervals; RMSSD, root mean square difference of successive RR intervals; pNN50, portion of adjacent varying by >50 ms; VLF, very low frequency spectral analysis of RR intervals; LF, low frequency spectral analysis of RR intervals; HF, high frequency spectral analysis of RR intervals; SD1, standard deviation of data from the long axis of the Poincare plot; SD2, standard deviation of the data from the axis perpendicular to the long axis of the Poincare plot. ^†^, significant main effect of time (*p* < 0.05); ^‡^, significant time × group interaction effect (*p* < 0.05).

## Data Availability

Data are available from the corresponding author, upon reasonable request.

## References

[1] White A., Ernst E. (2004). A brief history of acupuncture. Rheumatology.

[2] Longhurst J.C. (2010). Defining Meridians: A Modern Basis of Understanding. J. Acupunct. Meridian Stud..

[3] World Health Organization (2007). WHO International Standard Terminologies on Traditional Medicine in the Western Pacific Region.

[4] Spaulding K., Chamberlin K. (2011). The Transport of Extremely Low-Frequency Electrical Signals Through an Acupuncture Meridian Compared to Nonmeridian Tissue. J. Altern. Complement. Med..

[5] Ahn A.C., Wu J., Badger G.J., Hammerschlag R., Langevin H.M. (2005). Electrical impedance along connective tissue planes as-sociated with acupuncture meridians. BMC Complement. Altern. Med..

[6] Chen K.-G. (1996). Electrical properties of meridians. IEEE Eng. Med. Biol. Mag..

[7] Lee M.S., Jeong S.Y., Lee Y.H., Jeong D.M., Eo Y.G., Ko S.B. (2005). Differences in electrical conduction properties between me-ridians and non-meridians. Am. J. Chin. Med..

[8] Jo H.-G., Jo G.-H. (2011). Effect of Acu-Magnetic Stimulation on Heart Rate Variability. Med. Acupunct..

[9] Jo H.-G., Jo G.-H. (2011). Electroencephalogram activity induced by magnetic stimulation on heart meridian. Neurosci. Lett..

[10] Shaffer F., McCraty R., Zerr C.L. (2017). A healthy heart is not a metronome: An integrative review of the heart’s anatomy and heart rate variability. Front Psychol..

[11] Del Paso G.A.R., Langewitz W., Mulder L.J.M., Van Roon A., Duschek S. (2013). The utility of low frequency heart rate variability as an index of sympathetic cardiac tone: A review with emphasis on a reanalysis of previous studies. Psychophysiology.

[12] World Health Organization (2008). WHO Standard Acupuncture Point Locations in the Western Pacific Region.

[13] Niskanen J.P., Tarvainen M.P., Ranta-Aho P.O., Karjalainen P.A. (2004). Software for advanced HRV analysis. Comput. Methods Programs Biomed..

[14] Tarvainen M.P., Niskanen J.-P., Lipponen J.A., Ranta-Aho P.O., Karjalainen P.A. (2014). Kubios HRV—Heart rate variability analysis software. Comput. Methods Programs Biomed..

[15] Pumprla J., Howorka K., Groves D., Chester M., Nolan J. (2002). Functional assessment of heart rate variability: Physiological basis and practical applications. Int. J. Cardiol..

[16] Vanderlei L.C.M., Pastre C.M., Hoshi R.A., Carvalho T.D., Godoy M.F. (2009). Basic notions of heart rate variability and its clinical applicability. Rev. Bras. Cir. Cardiovasc..

[17] Task Force of The European Society of Cardiology and the North American Society of Pacing and Electrophysiology (1996). Heart rate variability: Standards of Measurement, Physiological Interpretation and Clinical Use; Task Force of the European Society of Cardiology and the North American Society of Pacing and Electrophysiology. Circulation.

[18] Malliani A., Pagani M., Lombardi F., Cerutti S. (1991). Cardiovascular neural regulation explored in the frequency domain. Circulation.

[19] Pagani M., Montano N., Porta A., Malliani A., Abboud F.M., Birkett C., Somers V.K. (1997). Relationship Between Spectral Components of Cardiovascular Variabilities and Direct Measures of Muscle Sympathetic Nerve Activity in Humans. Circulation.

[20] Brown T.E., Beightol L.A., Koh J., Eckberg D.L. (1993). Important influence of respiration on human R-R interval power spectra is largely ignored. J. Appl. Physiol..

[21] Schipke J.D., Pelzer M., Arnold G. (1999). Effect of respiration rate on short-term heart rate variability. J. Clin. Basic Cardiol..

[22] Billman G.E. (2013). The LF/HF ratio does not accurately measure cardiac sympatho-vagal balance. Front. Physiol..

[23] Ahn A.C., Colbert A.P., Anderson B.J., Martinsen Ø.G., Hammerschlag R., Cina S., Wayne P.M., Langevin H.M. (2008). Electrical properties of acupuncture points and meridians: A systematic review. Bioelectromagnetics.

[24] Ahn A.C., Park M., Shaw J.R., McManus C.A., Kaptchuk T.J., Langevin H.M. (2010). Electrical impedance of acupuncture me-ridians: The relevance of subcutaneous collagenous bands. PLoS ONE.

[25] Qiu C., Zhao T., Li Q., Wang X., Xiao K., Wang B. (2021). A Low-Power Stable Wideband Current Source for Acupuncture Point Skin Impedance Measurements. J. Health Eng..

[26] Chang S.-A., Weng Y.-X., Cheng S.-C., Chang Y.-J., Lee T.-H., Chang C.-H., Chang T.-Y., Huang K.-L., Liu C.-H., Hsu C.-Y. (2019). Application of Meridian Electrical Conductance in the Setting of Acute Ischemic Stroke: A Cross-Sectional Study. Evid. Based Complement. Altern. Med..

